# Effect of a telerehabilitation exercise program versus a digital booklet with self-care for patients with chronic non-specific neck pain: a protocol of a randomized controlled trial assessor-blinded, 3 months follow-up

**DOI:** 10.1186/s13063-023-07651-z

**Published:** 2023-09-28

**Authors:** Juliene Corrêa Barbosa, Josielli Comachio, Amelia Pasqual Marques, Bruno Tirotti Saragiotto, Mauricio Oliveira Magalhaes

**Affiliations:** 1 Master’s Program in Human Movement Sciences, Federal University of Pará, Belém-Pará, 66050-160 Brazil; 2 School of Health Sciences, Faculty of Medicine and Health, Sydney Musculoskeletal HealthCharles Perkins CentreUniversity of Sydney, Sydney, NSW 2009 Australia; 3 Department of Physiotherapy, Speech-Language Pathology and Audiology and Occupational Therapy, Faculty of Medicine, University of São Paulo, Rehabilitation Sciences Program, São Paulo, 05360-160 Brazil; 4 Master’s and Doctoral Programs in Physical Therapy, Universidade Cidade de São, Paulo, São Paulo, 03071-000 Brazil; 5 Discipline of Physiotherapy, Graduate School of Health, University of Technology, Sydney, Sydney, Australia

**Keywords:** Neck pain, Chronic pain, Internet-based intervention, Clinical trial protocol

## Abstract

**Background:**

Neck pain is the fourth worldwide leading cause of disability and represents 22% of musculoskeletal disorders. Conservative intervention has been strongly recommended to treat chronic neck pain and Telerehabilitation is the alternative for the treatment of musculoskeletal conditions. There is a lack of high-quality research on the effects of telerehabilitation in patients with neck pain and functional disability. Therefore, this study aims to evaluate the effect of a telerehabilitation exercise program versus a digital booklet only with self-care information in individuals with non-specific chronic neck pain.

**Methods:**

This is a prospectively registered, assessor-blinded, two-arm randomized controlled trial comparing a telerehabilitation exercise program versus a digital booklet with self-care information. Seventy patients will be recruited with non-specific chronic neck pain. Follow-ups will be conducted post-treatment, 6 weeks, and 3 months after randomization. The primary outcome will be disability at post-treatment (6 weeks) measured using neck pain disability. Secondary outcomes will be pain intensity levels, global perceived effect, self-efficacy, quality of life, kinesiophobia, and adherence to treatment. In our hypothesis, patients allocated to the intervention group experience outcomes that are similar to those of those assigned to the self-care digital booklet. Our hypothesis can then be approved or disapproved based on the results of the study.

**Discussion:**

This randomized clinical trial will provide reliable information on the use of telerehabilitation to treat patients with chronic non-specific neck pain.

**Trial registration:**

The study was prospectively registered at the Brazilian Registry of Clinical Trials (number: RBR-10h7khvk). Registered on 16 September 2022.

## Introduction

Neck (cervical) pain is the fourth worldwide leading cause of disability and represents 22% of musculoskeletal disorders [1, 2]. It is estimated that neck pain affects more than 65 million people annually worldwide — annual incidence remains between 15 to 50% — and 70% of the population is affected throughout life [3]. The Global Burden of Disease 2017 study indicated neck pain as the ninth and eleventh cause of years lived with disability among women and men, respectively [4]. Overall, neck pain can result in significant health costs, including medical expenses, loss of productivity, and costs associated with various treatments. The average annual health costs for individuals with neck pain were US$ 3709 to US$ 2731 for those without neck pain [5]. In the Netherlands, total annual societal costs of neck pain were estimated at US$ 686 million [6].

Chronic non-specific neck pain is restricted to the cervical region without radiation to the upper limbs and symptoms last more than 12 weeks. It can be defined as the pain in the posterior cervical region from the superior nuchal line to the spine of the scapula and the side region down to the superior border of the clavicle and the suprasternal notch [7, 8].

Conservative intervention has been strongly recommended to treat chronic neck pain. Postural and mobility exercises, and cervical (or cervicothoracic) manipulation or mobilizations combined with stretching and strengthening have been found as evidence-based treatments to decrease neck pain and improve range of motion [9, 10]. Cervical exercise is an effective treatment for neck pain [11, 12]. A recent systematic review of patients with chronic neck pain concluded that multimodal training (exercises involving deep and superficial cervical muscles) is necessary to have beneficial effects on function and symptoms [13]. A recent systematic review with meta-analysis showed that motor control, Yoga, Pilates, TaiChi, and strengthening exercises have beneficial effects on neck pain relief when compared with no treatment [14].

Telerehabilitation is the use of communication and information technologies such as telephones, video conferencing, sensors, virtual reality, and robotics to deliver rehabilitation services at a distance [15, 16]. This practical modality is becoming popular among patients with musculoskeletal pain as a solution to healthcare access barriers related to travel conditions (distance, transit, transportation) and high demand (long waiting lists) [17–19]. Recent studies indicate that telerehabilitation can improve accessibility to healthcare services and increase patients’ adherence level to exercise programs due to its flexibility and convenience [20–22]. The feasibility of telerehabilitation protocols to treat hip and knee arthroplasty, non-specific low back pain, rheumatoid arthritis, osteoarthritis, and others have been reported [19, 23, 24]. Furthermore, telerehabilitation has been used in a wide range of fields including musculoskeletal pain [25, 26]. Then, high-quality randomized controlled trials are needed to investigate the effectiveness of telerehabilitation for people with neck pain.

A recent study observed that telerehabilitation is an effective method to improve function and relieve pain in patients with musculoskeletal conditions [27]. The only two studies on the effectiveness of telerehabilitation to treat patients with chronic non-specific neck pain have presented methodological flaws such as lack of evaluator blinding, intention-to-treat analysis, and hidden allocation [28, 29]; hence, studies that follow adequate methods are needed.

Therefore, this randomized controlled trial aims to evaluate the effect of a telerehabilitation exercise program versus a digital booklet only with self-care information (chronic pain, benefits of physical exercise, and healthy lifestyle guidelines) in individuals with non-specific chronic neck pain. The primary outcome will be disability at post-treatment (6 weeks) measured using neck pain disability. Secondary outcomes will be pain intensity levels, global perceived effect, self-efficacy, quality of life, kinesiophobia, and adherence to treatment.

## Methods

### Study design

This two-arm, randomized, controlled, and evaluator-blinded study will be conducted under both the Consolidated Standards of Reporting Trials (CONSORT) [30] and Standard Protocol Items: Recommendations for Interventional Trials (SPIRIT) [31].

### Register and protocol version

The Research Ethics Committee of the Federal University of Pará, Brazil (registration no. 5.458.454), granted approval for this clinical trial. The study adhered to the ethical principles stated in the Declaration of Helsinki for human studies. Additionally, the trial was registered with the Brazilian Registry of Clinical Trials (registration no. RBR-10h7khvk). All co-authors provided their approval for the study.

### Study setting and recruitment procedure

Physiotherapists from the outpatient musculoskeletal physiotherapy department at the Faculty of Physiotherapy and Occupational Therapy, Federal University of Pará, Belem, Brazil, will identify all patients who have been experiencing neck pain for more than 12 weeks and are seeking care for chronic neck pain. If patients express interest in participating, their contact details will be provided to the research team.

### Potential participants from the general community

To facilitate recruitment from the general community, the study advertising materials and documents will be shared through community channels and social media platforms. This will include, but is not limited to, promotion through social media and online advertising services. The social media strategy will involve sharing the study poster and the study webpage URL on platforms such as Facebook and Instagram.

### Eligibility criteria

Potential participants will be phoned to determine eligibility before enrollment:

- 18 to 60 years old male or female interested to treat chronic neck pain.
- Access to the internet via computer/smartphone.
- Read and understand the Portuguese language.
- Neck pain for more than 12 weeks [32].

Individuals with severe musculoskeletal disorders, cardiovascular and metabolic disorders, history of neurological injuries, use of muscle relaxants, obesity (body mass index > 30 kg/m^2^), red flags (unexplained weight loss, fever, moderate to severe trauma, among others) [33], cognitive problems, visual or hearing impairments, or any health condition that hinder safe and proper participation in online exercise sessions will be excluded.

The Physical Activity Readiness Questionnaire (PAR-Q) of the Canadian Society of Exercise Physiology will be used to determine the ability of participants to perform physical activities [34, 35].

### Procedure

**Fig. 1:**
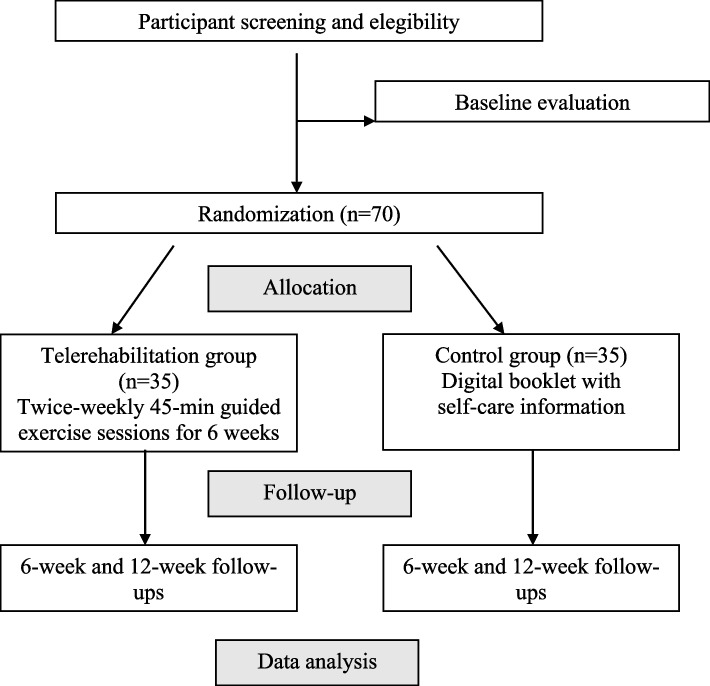
Study flowchart

Eligible participants will receive a digital informed consent form and will be informed about the study objectives by a blinded and trained evaluator. Participants will be referred for baseline assessment consisting of the demographic (age, sex, weight, height, marital state, profession, and education level), clinical (comorbidities, duration of pain, location of pain, average pain intensity, pain area) and medical data. The baseline evaluation will be performed via video conferencing, while the 6-week and 12-week follow-ups will be performed via phone call, smartphone text message, or email by using a Google Forms questionnaire (Fig. 1). All data will be encoded and typed twice using Microsoft Excel. A second blinded evaluator will double-check all data before analysis.

### Primary outcome

Disability will be measured through the Brazilian-Portuguese version of the Neck Disability Index [36], which is a 10-item self-administered questionnaire regarding limitations in daily activities due to neck pain. The total score ranges from 0 (no limitations) to 50 (major limitations) [36].

### Secondary outcomes

- Pain intensity levels will be measured through the 11-point Pain Numerical Rating Scale, which ranges from 0 (no pain) to 10 (worst possible pain) [37].
- Overall health transition will be measured through the 11-point Global Perceived Effect Scale, which ranges from − 5 (extremely worse) through 0 (no change) to + 5 (completely recovered). A higher score represents a better recovery from the condition [37].
- The self-efficacy in chronic pain will be measured through the 22-item Chronic Pain Self-efficacy Scale (CPSS). Each domain (self-efficacy for pain control (PSE)); self-efficacy for physical function (FSE); and self-efficacy symptoms control (CSE)) score ranges from 10 (very uncertain) to 100 (very certain) and the maximum total score of 300 points indicates the greatest sense of self-efficacy [38].
- Health-related quality of life will be measured by the 12-item revised Short-Form Health Survey (SF-12v2) on 7 multidimensional domains. A higher score indicates better quality of life [39].
- The avoidance of movements or activities based on fear will be measured through the 17-item Tampa Scale of Kinesiophobia (TSK) individually based on a 4-point Likert scale that ranges from “totally disagree,” through “partially disagree,” “partially agree,” and “totally agree.” Items 4, 8, 12, and 16 are reversed scored, and higher scores indicate greater kinesiophobia [40].

Participants will be evaluated at baseline, after 6 and 12 weeks. Patients allocated in the intervention group will be treated by a physical therapist specialized in traumatology and orthopedics and experienced in chronic pain treatment. This evaluator will confirm the eligibility of participants and will be blind to their allocations.

All instruments used were translated and adapted to Brazilian-Portuguese versions, with adequate psychometrical properties. Pain, disability, global perceived effect, self-efficacy in chronic pain, quality of life, and kinesiophobia will be measured [36–40].

### Randomization and group allocation

The Research Randomizer tool (http://www.randomizer.org/) will be used to generate random numbers and assign the participants to the Telerehabilitation Group and Control Group. Allocation (1:1 ratio) will be performed by an independent researcher without participation in data acquisition and statistical analysis. Concealed allocation will be performed by using sequentially numbered, opaque, and sealed envelopes. The same blinded evaluator will open the sealed envelopes after the informed consent form is filled and perform the participant baseline evaluation. Participants will receive a unique study registration number and will be referred to the physical therapist responsible for each group.

### Data collection and management plan

Participants will be identified by an individual trial number (ID) to ensure confidentiality, coding, and confidentiality. A data management plan will also be designed in accordance with the recommendations and regulations of the Federal University of Pará, Belem, Brazil. The security and confidentiality of the data collected at all stages will be ensured by storing it on password-protected servers. Paper-based data will be kept in locked filing cabinets at the Postgraduate Program in Human Movement Sciences. Access to the data will be restricted to the lead investigator only. All statistical analyses will be performed using the individual number of each participant and the statistician will be blinded to the group. The results will be presented by group data and any individual data will be spread to ensure confidentiality is preserved.

A Data Monitoring Committee (DMC) will be convened to overview data collection and integrity. The DMC will approve the statistical analysis plan and research protocol. The integrity of trial data will be monitored by regularly scrutinizing data sheets for omissions and errors. Data inconsistencies will be explored and resolved. The lead investigator will be responsible for overseeing trial safety and ensuring that the best interests of participants are observed at all times. The lead investigator will be blinded to allocation, unless unblinding is deemed essential to ensure participant safety. Adverse events will be reported to the reviewing Human Research Ethics Committee and approved requirements.

An adverse incident is defined as a harmful, unpleasant, or undesirable response, reaction, or outcome experienced by a research participant or researcher. Adverse events that may be expected as part of the interventions or usual care that do not need to be reported to the HREC include, muscle soreness, swelling, or muscle cramps related to commencement of unaccustomed exercise or trips and/or falls, that have not resulted in an injury. A serious adverse event is defined as an event that may result in death, be life-threatening, require or prolong inpatient hospitalization, result in persistent or significant disability/incapacity, is a congenital anomaly or birth defect, is some other important medical event, and is expected or unexpected.

The collected data will be accessible only to the lead investigator and a few designated members of the research team. Since the intervention is deemed safe and poses minimal risk to participants, there may not be a requirement for interim analysis in this study. However, a safety monitoring plan will be strictly adhered to as outlined.

### Study blinding

The evaluator will be blinded to the participant’s randomization and allocation sequence. Blinding of participants and therapists will not be possible due to the nature of the study.

### Telerehabilitation group

Participants will follow twice a week, 45-min exercise sessions for 6 weeks via video conferencing guided by a 2-year experienced physiotherapist specialized in traumatology and orthopedics. Participants will receive phone text messages to schedule individual exercises. The patients will be instructed to wear light and comfortable clothes during the exercise sessions.

The telerehabilitation program will be divided into different phases with different goals for the participants. The first phase of the exercise program (first and second weeks) will focus on gaining mobility. The 2nd phase (third and fourth weeks) will focus on cervical and central stabilization. The 3rd phase (fifth and sixth weeks) will focus on muscular resistance improvement of superior and inferior limbs. The descriptions of all the exercises are in Table 1. Exercise will progress by varying the repetitions, load, and a self-rated effort level of at least 5 out of 10 (hard) on a modified Borg Rating of Perceived Exertion scale [41]. In the fifth and sixth weeks, exercise series of 12 to 15 repetitions is expected to be performed with 1 kg and 2 kg objects (dumbbells, water container, others), respectively [32, 42–45].

**Table 1:** Description of the telerehabilitation exercise program

Exercise	Description	Series/duration
Stretching (1ª phase)	Sitting position: - Close the hands underneath the base of the head and stretch the cervical flexors by pulling the head forward and down - Put hands under the chin and stretch the cervical extensors by pushing up - Flex the trunk and stretch erector spinal muscles by touching both hands on the ground Upright position: - Put the hand on the opposite head side and stretch the trapezius by hand pulling the head towards the shoulder (then the other hand)	3 series of 30 s with 1-min Interval
Mobilization (1ª phase)	Sitting position: - Actively mobilize the cervical region through flexion, rotation, lateral inclination, and extension movements - Lift the shoulders towards the ears and return to the initial position “Cat-cow” position supported by knees and hands: - Lift the thoracic spine, flex the cervical spine, put the head down, and return to the initial position	2 series of 10 repetitions with 5-s sustain and 1-min intervals
Cervical stabilization (2ª phase)	Sitting position: - Pull the head and chin back - Pull the head back and flex the neck Ventral decubitus position with forearms on the ground: - Extend the trunk and pull the head and chin back	2 series of 10 repetitions with 5-s sustain and 1-min interval
Central stabilization (2ª phase)	Dorsal decubitus position and feet flat on the ground: - Elevate the pelvis until the knees reach 90° - Elevate the pelvis until the knees reach 90° and stretch one leg (then the other leg)	2 series of 10 repetitions with 5-s sustain and 1-min interval
Upper limb resistance (3ª phase)	Upright position: - Hand hold dumbbells down and lift stretched arms forwards from the thighs up the shoulder level; - Hand hold dumbbells down and lift stretched arms forwards from the thighs up the shoulder level; - Flex/extend the elbows holding weight; - Flex elbows by lifting handhold dumbbells up to shoulder level	3 series of 15 repetitions with a 2-min interval
Lower limb resistance (3ª phase)	Sit and stand up from a chair with arms crossed	3 series of 15 repetitions with a 2-min interval

Physical therapists with expertise in traumatology and orthopedics and in the care of patients with spinal diseases carefully prepared the workout program based on the literature. To ensure a more assertive application, the exercise program was subjected to a round of suggestions and modifications from experts in the treatment of patients with spinal pain.

In addition to the exercise program, the participants will receive a digital booklet with self-care information about chronic pain, the benefits of physical exercise, and healthy lifestyle guidelines such as the importance of adequate sleep and nutrition to improve s quality of life.

An online attendance list will be used to track adherence to the exercise program and participants can clarify any doubts regarding the booklet.

### Control group

Participants will receive the same digital booklet with self-care information containing general information about self-management of chronic pain, including pain education, advice on healthy lifestyle and sleeping habits and promotion of physical activity. In addition, participants will receive phone text messages, WhatsApp messages, or e-mails once a week to encourage the maintenance of healthy habits during the study period. The participants will be able to clear doubts through telephone contact. The participants of both groups were instructed not to change any medication prescribed by their physician and not to seek other treatment for their low back pain during the study.

### Criteria for discontinuing adverse effects and adherence to interventions

The criteria for discontinuation is allowed once a participant requests explicitly or refuses to continue the treatment or follow-up assessment, and the reason will be fully reported. The study will be discontinued in case of serious adverse events (any significant disability, hospitalization, life-threatening, and death) occur that make continuing the study harmful for the participants regardless of if related to the intervention (or control) or not. Ancillary and post-trial care (e.g., provision and/or cover for additional health care of immediate adverse events related to trial procedures) will be provided for participants who suffer sustained harm because of their involvement in this trial at no cost. Even though the study is considered low-risk, patients are encouraged to contact the research team in case of doubts or adverse effects. Moreover, the adherence intervention will be assessed by sending appointment text reminders 30 min ahead of each appointment.

### Plans for communicating protocol amendments to relevant parties and trial results to participants

Important protocol modifications such as changes to eligibility criteria, outcomes, or analyses will be notified to relevant parties (e.g., Research Ethics Committee, researchers, participants, and journal of publication). Furthermore, the results of the trial will be presented to the participants by email or in future scientific publications.

### Sample size calculation

The sample size and power calculations were based on the previous study [46]. The calculations were based on detecting a mean difference of 4.21 points on the Neck Disability Index, assuming a standard deviation of 5.58 points, a two-tailed test, an alpha level of 0.05, a desired power of 80% and an estimated 15% dropout rate. These assumptions generated a sample size of a minimum of 32 participants per group.

### Statistical analysis

The data distribution will be determined by visual analysis of histograms. The baseline characteristics of participants will be calculated through descriptive statistics. Potential differences between groups and 95% confidence interval for the 6-week and 3-month follow-ups after randomization will be calculated by using Linear Mixed Models with the interaction between the intervention group and time. Missing data will be handled using linear mixed models (ie, imputation methods were not needed) [47]. An estimation approach will be used to interpret the findings rather than using statistical significance. The principles of intention-to-treat analysis will be used [48] and data analysis will be performed by using the Statistical Package for the Social Sciences (SPSS) version 17.0.

## Discussion

This randomized clinical trial aims to present a protocol to investigate the effect of a telerehabilitation program versus a self-care booklet on pain and functional disability, through a randomized controlled trial designed for patients with chronic nonspecific neck pain. The null hypothesis tested will be that patients allocated in the intervention group present outcomes not significantly different from the control group. In our hypothesis, patients allocated to the intervention group experience outcomes that are similar to those of those assigned to the self-care digital booklet. Our hypothesis can then be approved or disapproved based on the results of the study.

Neck pain is a common musculoskeletal condition that impairs the life quality of millions of people worldwide [49] and is a clinically important condition for physical therapists. This randomized clinical trial will provide reliable information on the use of telerehabilitation to treat patients with chronic non-specific neck pain. It is expected that telerehabilitation of patients with neck pain by using an exercise program will figure as an efficient method.

There is a lack of high-quality research on the effects of telerehabilitation in patients with neck pain and functional disability. Therefore, the outcomes of this study will corroborate the scientific community on the effectiveness of a telerehabilitation protocol in patients with neck pain.

## Data Availability

All data will be used only for analysis of the present study and will be protected from any unnecessary exposure. The Consent Form according to the Research Ethics Committee of Universidade Federal do Pará was signed by the authors and patients. The paper information will be kept in binders that will be handled only by the researchers responsible for the study and will be kept in locations that will be accessible only to researchers responsible for data analysis. The online information will be able to the researchers on a personal computer located in the personal room of the researcher responsible for the project. All online information will be assessed just by the responsible researchers of the specific part of the data analysis. All information will be published confidentially, without the name of the subjects exposed. All data will be available for review and confirmation of data analysis when requested by a review process for publication of the article in indexed scientific journals or presentations at the scientific event.

## Abbreviations

SPIRIT: Standard Protocol Items—Recommendations for Interventional Trials
CONSORT: Consolidated Standards of Reporting Trials
DMC: Data Monitoring Committee
